# Effect of the internet combined with exercise-based individualized nursing intervention in patients with gestational diabetes mellitus

**DOI:** 10.1186/s13098-021-00738-0

**Published:** 2021-10-30

**Authors:** Yaer Chen, Chunbo Qiu, Jie Chen, Lu Li, Jichao Xu, Zhiren Sheng

**Affiliations:** 1 Department of Obstetrics, Affiliated Hospital of Ningbo University School of Medicine, Ningbo, 315000 Zhejiang China; 2 Department of Nursing, Affiliated Hospital of Ningbo University School of Medicine, No. 247, Renmin Road, Ningbo, 315000 Zhejiang China

**Keywords:** Internet combined with exercise-based individualized nursing, Gestational diabetes mellitus, Glucose tolerance, Insulin resistance

## Abstract

**Background:**

Gestational diabetes mellitus (GDM) is the most frequent medical complication of pregnancy. This condition is manifested by glucose intolerance resulting in hyperglycemia of variable severity during pregnancy. One of the most important clinical tools for efficiently regulating maternal blood glucose is strictly monitoring blood glucose levels. However, due to a lack of appropriate intervention tools, managing the occurrence of GDM is still unfeasible. This study aimed to determine clinical efficacy of the internet combined with exercise-based individualized nursing intervention in patients with gestational diabetes mellitus (GDM).

**Methods:**

In total, 139 patients with GDM were divided into two groups, with 79 patients in the observation group (internet combined with exercise-based individualized nursing intervention) and 60 patients in the control group (routine nursing intervention only). The two groups were given specified nursing intervention for 8 weeks and then compared for changes in their blood glucose, blood lipids, blood pressure, insulin resistance (IR), and rate of adverse pregnancy outcomes. Additionally, the psychological state was analyzed, and their nursing satisfaction with the care from nurses that they received was investigated before and after the nursing intervention.

**Results:**

Compared with the control group, the following indices of the observation group were lowered: blood glucose-related indices (FBG and 2 h PG), blood lipids and blood pressure associated indices (TG, TC, and HbA1c, DBP, SBP, and MAP), and IR-related indices (FINS, 2 h INS, and HOMA -IR) (all P < 0.05). The observation group also showed a lower rate of adverse pregnancy outcomes than the control group (7.59% *vs.* 20.00%; P < 0.05). In addition, SAS and SDS scores of the observation group were both lower than the control group (P < 0.05). Accordingly, the nursing satisfaction score also displayed that the observation group (93.67%) had a higher satisfaction outcome than the control group (76.67%; P < 0.05).

**Conclusions:**

Internet combined with exercise-based individualized nursing intervention in GDM patients can effectively improve their blood glucose, IR, and psychological status, thus significantly improving their pregnancy outcomes and mental condition.

## Introduction

Gestational diabetes mellitus (GDM) is defined as carbohydrate intolerance that begins or manifests during pregnancy [1]. GDM disrupts 18% of pregnant women globally, and most of them suffer from GDM due to impaired glucose tolerance (GT) during pregnancy [2]. Women with GDM face a higher risk of hypertension during pregnancy and are more likely to have early birth. GDM is linked to a number of fetal problems, including macrosomia, neonatal hypoglycemia, congenital abnormalities, and prenatal death [3, 4]. GDM is also associated with an increased risk of developing type 2 diabetes in the mother [5]. To better ensure the health and safety of both mother and fetus, it is imperative to strictly control the blood glucose levels of pregnant women during pregnancy [6]. In clinical practice, pregnant women are generally recommended to do more exercise and follow a more regular diet once they are found to have high blood glucose, and in some cases, they are advised to take drugs if they still cannot reach the targeted blood glucose levels [7]. However, long-term administration of drugs adversely affects both mother and the fetuses [8]. Therefore, effective intervention measures and scientific guidance are safe and recommended therapy to lower the blood glucose level of pregnant women.

Previous studies have shown that nursing intervention can help alleviate the GDM of patients [9, 10]. Lv et al. [11] suggested the positive role of comprehensive nursing intervention in the pregnancy outcome of patients with GDM, which implies the importance of nursing intervention to patients with GDM. The internet combined with nursing services is a novel, innovative nursing mode proposed in China in recent years [12]. Under such a nursing mode, patients can enjoy professional guidance and get help from the medical staff at home anytime, which is a favorable treatment for those who require effective continuous treatment and nursing [13]. In addition, exercise-based individualized nursing is a new nursing mode under which nurses provide various comprehensive services to the patients [14]. Some studies have pointed out the significant effect of exercise-based individualized nursing in patients with type 2 diabetes mellitus in clinical practice [15, 16]. However, the application of the internet combined with exercise-based individualized nursing intervention in patients with GDM is not well defined. Therefore, this study analyzed the effect of such a combined nursing intervention on glucose tolerance (GT) and insulin resistance (IR) in patients with GDM to offer an effective tool and guidance for future clinical nursing intervention for GDM.

## Methods

### Participants

A total of 139 patients with GDM admitted to our hospital between July 2019 and February 2021 were enrolled in this study and randomly divided into two groups according to the numerical random table method. Among them, 79 patients were given the internet combined with exercise-based individualized nursing intervention as the observation group, while the rest were given routine nursing intervention only as the control group. This study was carried out with permission from the Ethics Committee of our hospital (Approval number: 2019-03G7002) and informed consent forms signed by all enrolled individuals.

Inclusion criteria were as follows: Age > 20; Patients meeting the diagnostic criteria of GDM according to the examination results in our hospital [17]; patients who were pregnant for the first time; patients with detailed case data; and those who or whose legal guardian signed informed consent forms based on voluntary participation.

The exclusion criteria were based on: Patients with comorbid chronic diseases; cardiovascular or cerebrovascular diseases or organ dysfunction; patients with drug allergies; consciousness disturbance or communication obstacle; and those with multiple pregnancies.

### Nursing intervention

The control group was given routine nursing intervention. Specifically, the nursing staff was arranged to pay close attention to the patients' blood glucose and blood pressure levels, inform the patients and their families of daily precautions such as diet adjustment and moderate exercise. If necessary, the staff was required to urge the patients to take drugs according to the doctor's advice and call back the patients to understand their basic situation and provide corresponding guidance according to their condition.

The observation group was given the internet combined with exercise-based individualized nursing intervention. In brief, patients and their families were enabled to make an online appointment to raise questions and obtain answers through a network platform provided by the hospital. Specialists and nurses would formulate targeted nursing intervention measures according to each patient's own situation. At discharge time, each patient was instructed to receive reminders about daily precautions and exercise on the internet on time. In addition, the nursing staff were arranged to post guidance on psychology, physiology, and blood glucose control on the internet, and to pay real-time attention to the patients' requirements and blood glucose levels and thus take corresponding adjustment measures on time to ensure the continuity of patients' treatment in hospital and at home. Moreover, the patients were encouraged to maintain a good state of mind and were given practical assistance in lowering blood glucose. The staff was also arranged to design targeted exercise schemes for patients according to their gestational age, such as simple brisk walking, walking with raised arms, and maternity exercises. Patients were advised to stop the exercise when they had slight sweating. The patients were reminded to take a walk every day, 30 min each time, under the premise of ensuing heart rate < 120 times /min after exercise. Furthermore, the nursing staff was required to emphasize safety precautions to each patient before exercise and help patients avoid dangerous and violent actions in time to protect pregnant women and fetuses from being hurt. During exercise, timely water replenishing was necessary to prevent hypoglycemia.

The patients in both groups received the specified nursing intervention for 8 weeks, and the parameters were measured before the intervention and at 37 weeks gestation.

### Assessment of blood glucose and other measurements

#### Blood glucose detection

Venous blood was sampled from each patient in the two groups, and their fasting blood glucose (FBG) and 2-h postprandial blood glucose (2 h PG) levels were measured via an automatic blood glucose analyzer.

#### Blood lipid detection

An automatic biochemistry analyzer was adopted for the determination of changes in patients' triglyceride (TG), total cholesterol (TC), glycated haemoglobin (HbA1c), low-density lipoprotein—cholesterol (LDL-C) and high-density lipoprotein—cholesterol (HDL-C) levels.

#### Blood pressure measurement

An ambulatory blood pressure detector was used for measuring changes in patients' systolic blood pressure (SBP), diastolic blood pressure (DBP), and mean arterial pressure (MAP).

#### IR detection

The automatic biochemistry analyzer was used for measuring the fasting insulin (FINS), 1 h postprandial insulin (1 h INS), and 2 h INS, and then the homeostatic model assessment (HOMA)-IR index was calculated.

### Outcome measures

The self-rating depression scale (SDS) [18] and self-rating anxiety scale (SAS) [19] were used to assess the patients' psychological state. SDS and SAS are the commonly used norm-referenced scales. Both are 20 item Likert scales, in which items tap psychological and physiological symptoms and are rated by respondents according to how each applied to them within the past week, using a 4-point scale ranging from 1 (none, or a little of the time) to 4 (most, or all of the time) [20].

Adverse pregnancy outcomes were measured by evaluating the incidence of risk events such as uterine pregnancy, polyhydramnios, and premature delivery.

Patients' nursing satisfaction was assessed by a self-made nursing satisfaction questionnaire of our hospital. The questionnaire had a total score of 100 points, with a score above 90 points indicating high satisfaction, a score between 71 and 90 points indicating moderate satisfaction, a score between 51- and 70-points indicating improvement requirement, and a score below 50 points denoting dissatisfaction. The overall satisfaction was calculated as follows = (number of patients with high satisfaction + number of patients with moderate satisfaction)/ total number of patients × 100%.

### Statistical analysis

The data was analyzed using SPSS statistical software version 20 (IBM), and figures were drawn using Graphpad7. Enumeration data, expressed as a rate, was compared between the two groups using the chi-square test. Measurement data, expressed as the mean ± SD, was compared between the groups via the t-test. P < 0.05 value was considered as statistically significant.

## Results

### Baseline data comparison

When compared the clinical baseline data of the two groups of patients and various indicators before the nursing intervention, no statistical differences were seen between the two groups (P > 0.05) (Table 1).

**Table 1:** Comparison of clinical baseline data between the two groups before nursing intervention

	Observation group	Control group	t or χ^2^	P
Age	29.1 ± 4.7	28.6 ± 4.5	0.633	0.528
Gestational week	14.92 ± 5.93	15.24 ± 6.37	0.305	0.761
TG (mmoI/L)	3.14 ± 0.62	3.12 ± 0.65	0.185	0.854
TC (mmoI/L)	6.84 ± 0.52	6.91 ± 0.53	0.780	0.437
HbA1c (%)	9.14 ± 0.74	9.22 ± 0.81	0.606	0.546
LDL-C (mmoI/L)	2.41 ± 0.28	2.43 ± 0.31	0.398	0.691
HDL-C (mmoI/L)	3.84 ± 0.34	3.81 ± 0.30	0.542	0.589
DBP (mmHg)	114.62 ± 8.42	115.14 ± 8.19	0.365	0.716
SBP (mmHg)	142.63 ± 10.54	144.94 ± 9.86	1.316	0.191
MAP (mmHg)	110.14 ± 7.63	110.06 ± 8.34	0.059	0.953
FINS (mU/L)	22.81 ± 3.81	22.56 ± 4.04	0.373	0.710
1 h INS (mU/L)	114.63 ± 22.86	112.94 ± 25.16	0.413	0.680
2 h INS (mU/L)	48.44 ± 6.96	49.17 ± 7.53	0.591	0.555
HOMA-IR	4.89 ± 0.32	4.93 ± 0.30	0.750	0.455
Family history of illness			0.823	0.364
Have	36	32		
No	43	28		
Place of residence			1.161	0.281
town	64	44		
countryside	15	16		
Nationality			1.795	0.180
Han nationality	74	59		
minority	5	1		
Education			0.142	0.707
> High School	22	15		
≤ High school	57	45		

### Blood glucose outcomes

Before nursing intervention, there was no significant difference in the FBG and 2 h PG levels between the control and observation groups (P > 0.05) (Table 1); however, after nursing intervention, a significant reduction in both FBG and 2 h PG levels was observed in the two groups (P < 0.05). Notably, a substantial improvement was seen in both FBG and 2 h PG levels in the observation group after nursing intervention compared to the control group (P < 0.05) (Table 2).

**Table 2:** Comparison of blood glucose levels in the two groups before and after nursing intervention (mmoI/L)

	Before nursing intervention		After nursing intervention	
	FBG	2 h PG	FBG	2 h PG
Observation group	7.24 ± 0.68	9.12 ± 0.59	5.56 ± 0.54^*#^	6.81 ± 0.67^*#^
Control group	7.26 ± 0.74	9.09 ± 0.63	6.98 ± 0.59^*^	8.12 ± 0.72^*^
t	0.165	0.774	14.750	11.060
P	0.869	0.288	< 0.001	< 0.001

(*) indicates a significant difference for P < 0.05 between before and after the nursing intervention. (#) indicates a significance for P < 0.05 between the control and observation groups

### Glucose tolerance outcomes

**Fig. 1:**
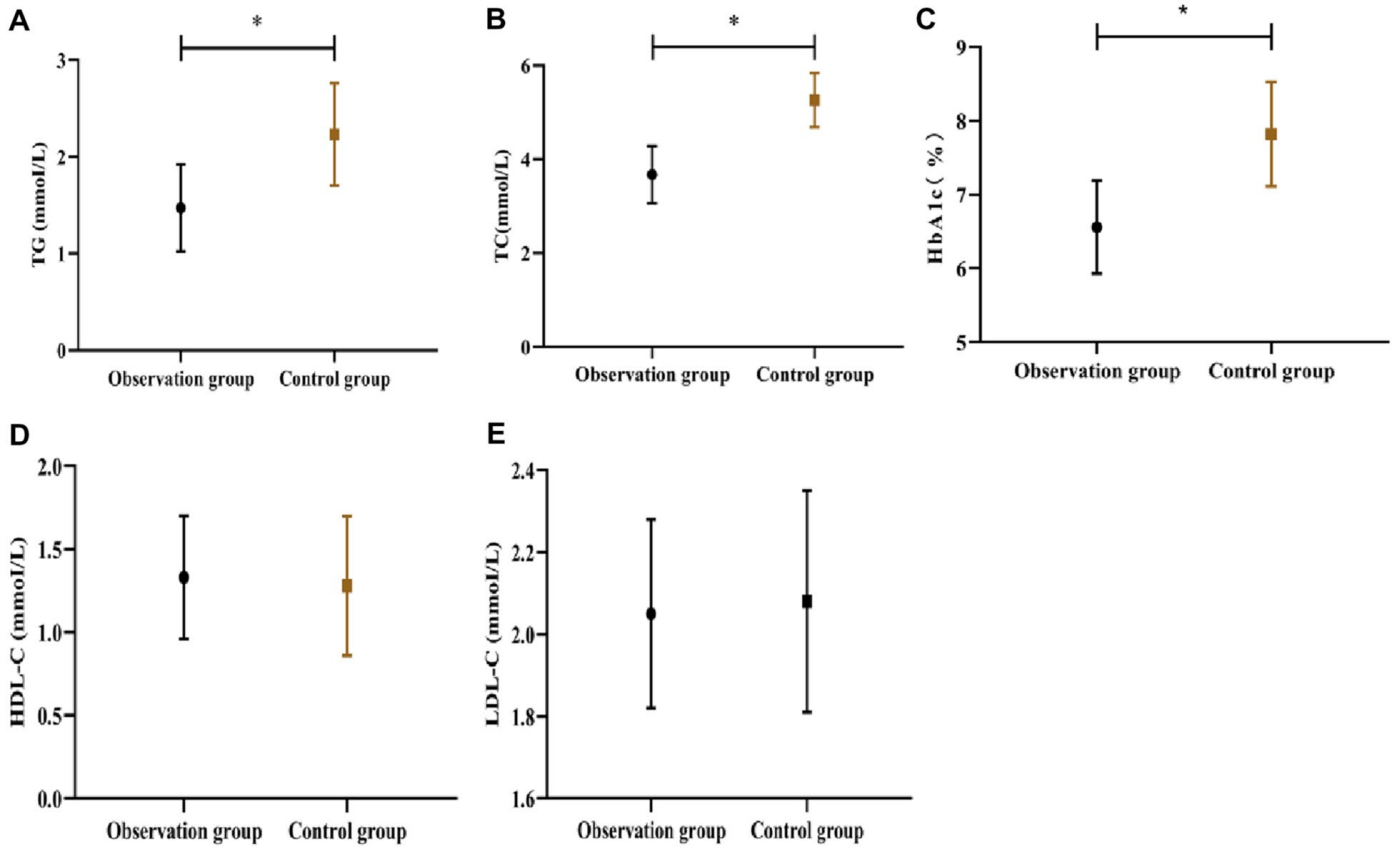
Comparison of glucose tolerance between the two groups. **A** Comparison of TG level. **B** TC level. **C** HbA1c level. **D** HDL-C level. **E** LDL-C level. The asterisk (*) indicates significance for P < 0.05 between the control and observation groups after nursing intervention

No prominent difference was seen for blood glucose tolerance between the two groups before nursing intervention (P > 0.05; Table 1). After nursing intervention, the levels of TG, TC, and HbA1c in the observation group were prominently lower than those in the control group (P < 0.05) (Fig. 1A–C), whereas no notable difference was found between the two groups in the LDL-C and HDL-C levels (P > 0.05) (Fig. 1D, E).

### Blood pressure outcomes

**Fig. 2:**
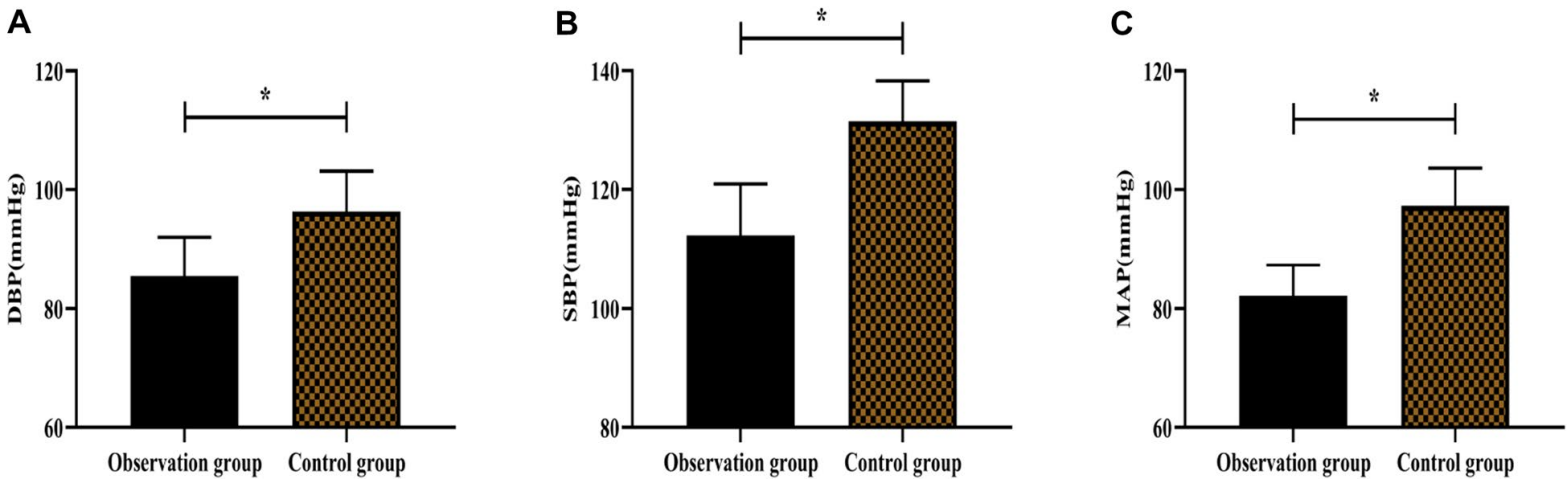
Comparison of the blood pressure between the two groups. **A** Comparison of DBP. **B** SBP. **C** MAP. The asterisk (*) indicates significance for P < 0.05 between the control and observation groups after nursing intervention

For blood pressure outcomes, no significant change was observed between the two groups before nursing intervention (P > 0.05) (Table 1). In contrast, as shown in Fig. 2A, the observation group had a reduced level of DBP compared to the control group after nursing intervention. Similarly, significantly lower levels of SBP and MAP were noticed in the observation group compared to the control group (Fig. 2B, C) (P < 0.05).

### Insulin resistance outcomes

**Fig. 3:**
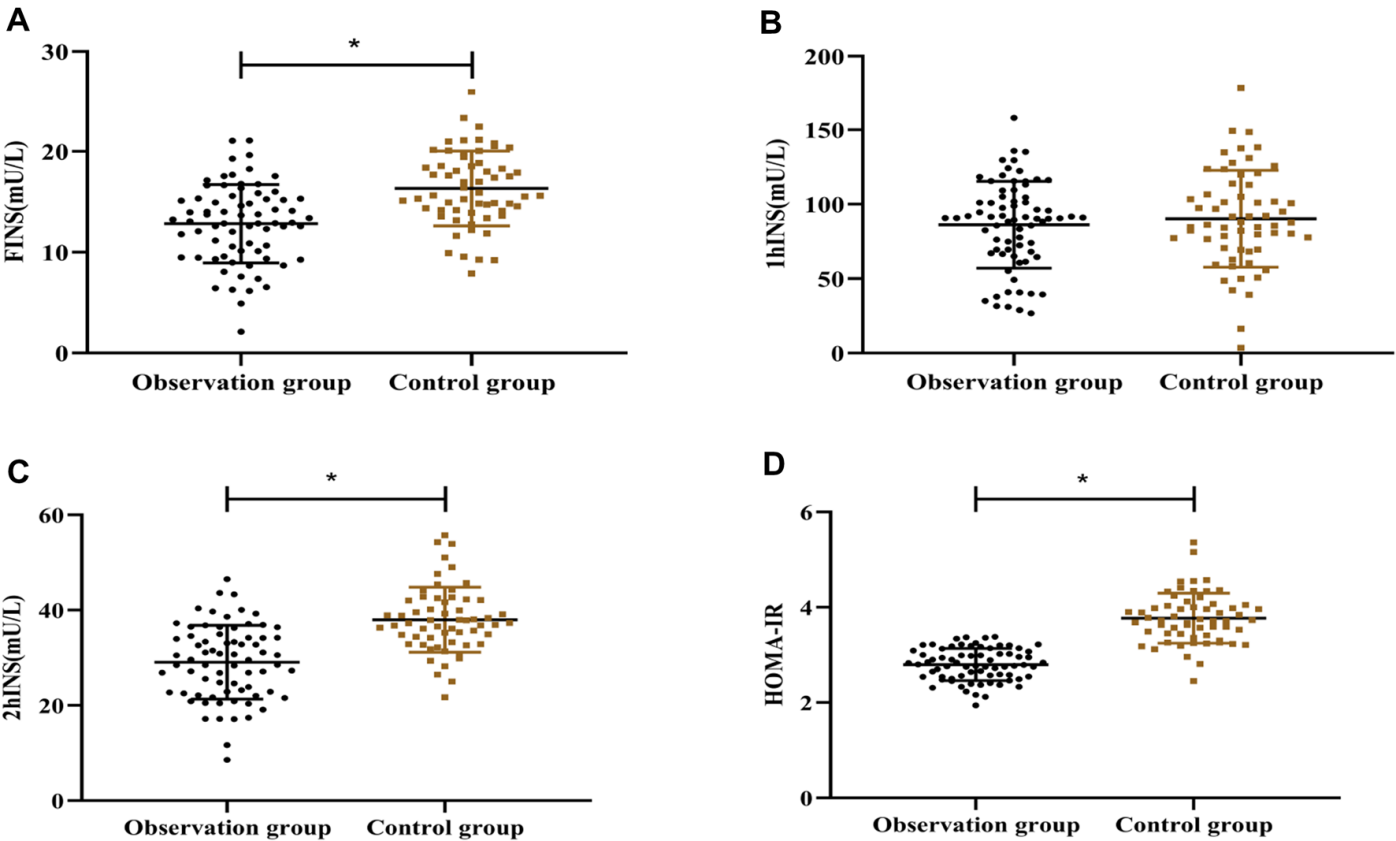
Comparison of insulin resistance between the two groups. **A** Comparison of FINS. **B** 1 h INS. **C** 2 h INS. **D** HOMA-IR. The asterisk (*) indicates significance for P < 0.05 between the control and observation groups after nursing intervention

Before the nursing intervention, no considerable change was noted for the levels of FINS, 1 and 2 h INS and HOMA-IR between the control and observation groups (P > 0.05) (Table 1). After nursing intervention, the two groups were not greatly different in the 1 h INS levels (P > 0.05) (Fig. 3B), but the FINS, 2 h INS and HOMA-IR levels of the observation group were all lower than those in the control group (P < 0.05) (Fig. 3A, C and D).

### Adverse pregnancy outcomes

The observation group showed a rate of adverse pregnancy outcome of 7.59% with 3 cases of cesarean section, 1 case of polyhydramnios, and 2 cases of fetal distress. In contrast, the control group displayed an adverse pregnancy outcome of 20.00% with 6 cesarean section cases, 3 cases of polyhydramnios, and 2 cases of fetal distress. The difference was statistically significant when the observation group was compared to the control group (P < 0.05) (Table 3).

**Table 3:** Comparison of adverse pregnancy outcomes

	Cesarean section	Polyhydramnios	Fetal distress	Premature delivery	Rate of adverse pregnancy outcome
Observation group	3 (3.80%)	1 (1.27%)	2 (2.53%)	0 (0.00%)	6 (7.59%)
Control group	6 (10.00%)	3 (5.00%)	2 (3.33%)	1 (1.67%)	12 (20.00%)
χ^2^					4.655
P					0.031

### Psychological state outcomes before and after nursing intervention

**Fig. 4:**
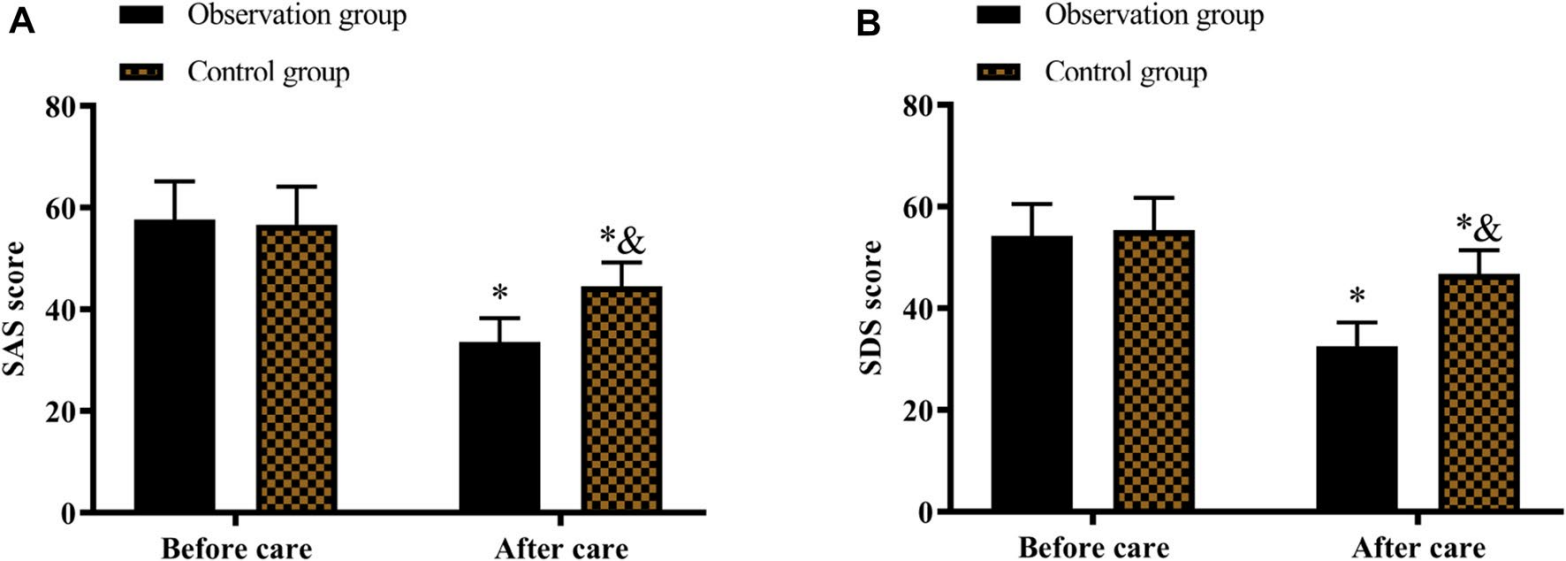
Comparison of psychological state between the two groups. **A** Comparison of SAS score. **B** SDS score. The asterisk (*) indicates significance for P < 0.05 between before and after care groups. (&) indicates significance for P < 0.05 between the control and observation groups after nursing intervention

Before nursing intervention, no noticeable differences were seen in the SAS and SDS scores between the two groups (P > 0.05). However, a significant change was observed in both groups' SAS and SDS scores after nursing intervention compared to before nursing intervention (P < 0.05). Of note, the SAS and SDS scores in the observation group were substantially improved compared to the control group after nursing intervention (P < 0.05) (Fig. 4).

### Nursing satisfaction

According to the investigation on nursing satisfaction, the observation group expressed a notably higher satisfaction score than the control group (93.67% *vs* 71.67%), and the difference was considerably high (P < 0.05) (Table 4).

**Table 4:** Comparison of nursing satisfaction between the two groups

	Very satisfied	Moderate satisfied	Room for improvement	Dissatisfied	Nursing satisfaction
Observation group	58 (73.42%)	16 (20.25%)	4 (5.06%)	1 (1.27%)	74 (93.67%)
Control group	19 (31.67%)	27 (45.00%)	11 (18.33%)	3 (5.00%)	46 (76.67%)
χ^2^					8.355
P					0.004

## Discussion

The results of this study supported the positive effects of the internet combined with exercise-based individualized nursing intervention in patients with gestational diabetes mellitus. It reduced blood pressure, blood lipids level, and most importantly, it significantly lessened the high blood glucose levels and adverse pregnancy outcomes of the GDM patients. In addition, it also positively modulated patients' psychological state and enhanced patients' satisfaction with the services provided by the nurses.

Insulin resistance (IR) and glucose tolerance (GT) are the main pathological bases of GDM [21]. Strictly monitoring blood glucose levels is one of the crucial clinical means for effectively controlling maternal blood glucose [22]. However, due to the lack of effective intervention means, it is still unfeasible to effectively manage the occurrence of GDM. In recent years, thanks to the continuous improvement in nursing interventions, the high-quality nursing intervention has become one of the crucial modes for patients' recovery and health [23]. In agreement with the above findings, our study results displayed that the observation group had notably lower blood glucose levels, higher GT, and better blood pressure and IR than the control group. These results imply the remarkable impact of the internet combined with exercise-based individualized nursing intervention in improving patients' blood glucose with GDM. As we all know, the treatment of GDM requires a long cycle, and it is necessary to gradually improve the sugar metabolism level in the body through drugs [24]. In this process, patients may carry out some activities not conducive to blood glucose control due to the lack of medical and health-associated knowledge that greatly compromises GDM treatment [25]. Therefore, for patients with GDM, in addition to stable and effective blood glucose control, patient guidance to help them understand the related knowledge of blood glucose and pay attention to the changes in their glucose metabolism is also necessary [26].

In actual daily life, nursing staff are unlikely to accompany patients to guide them in seeking advantages and avoiding disadvantages all the time. Therefore, real-time guidance to patients has become one of the keys to the prognosis of GDM. The implementation of internet-based nursing can effectively address this situation. With society's modernization, the internet has become an inseparable part of people's lives [27]. It enables us to realize direct real-time communication between doctors and patients. Using this methodology, patients can get a timely and rapid response from the medical staff once they encounter unsolvable problems related to diseases. Medical staff can also provide the most professional solution and suggestions to the patients on time. It can greatly lower the adverse risks faced by patients with GDM. According to one earlier study, it has been shown that internet nursing can reduce the incidence of adverse events in patients with chronic diseases [28]. In our study, we also found that the implementation of internet-based nursing intervention significantly reduced adverse events in GDM patients, highlighting the significance of internet nursing in clinical application.

In patients with GDM, the increase in blood glucose and blood lipids due to obesity is one of the primary risk factors of GDM [29]. The most effective and safe way to reduce the risk of obesity is effective exercise. Moreover, proper exercise can improve the human body's metabolism, immune function, and cardiopulmonary ability. In the present study, the guidance and attention given to the GDM patients by introducing nursing intervention did not only help them to have better blood glucose metabolism but also guided them to have a better body function and a higher success rate of childbirth. Other studies have also confirmed the beneficial outcomes of exercise-based nursing intervention in many diseases such as chronic obstructive pulmonary disease [30], Parkinson's disease [31], colon cancer [32], osteoporosis [33] and others. The results of these studies are in complete agreement with those of our experimental data. In our study, the observation group had better pregnancy outcomes than the control group, and the reasons may also be consistent with our above analysis, which fully demonstrates that the internet combined with exercise-based individualized nursing intervention can yield positive results in the GDM patients.

Modern clinical nursing services are no longer limited to the patients' diseases only but also provides importance to the patients' psychological state. Many studies have shown that most patients usually feel fear, rebellion, and conflict with the medical staff because of the pain caused by the disease and the lack of knowledge related to the disease [34–36]. Such a negative psychological state may not only greatly compromise a patient's treatment compliance, resulting in tension between doctors and patients, but can also directly minimize the clinical treatment effect. Therefore, in modern nursing services, nursing staff are required to actively communicate with patients to reduce the communication gap between the doctors and patients and build up patients' confidence to overcome fear related to diseases. According to the results of this study, the SDS and SAS scores in the observation group were notably lower than those in the control group. Most importantly, the results of the two groups' nursing satisfaction imply that the internet combined with exercise-based individualized nursing intervention is beneficial to GDM patients' and it has a great application prospect in the clinical practice.

## Strengths and limitations

To date, there has been no evidence of interventions that provide GDM patients with exercise-based individualized nursing intervention via the internet, which is already being used by the health system for other purposes. As a result, its adoption would not incur a high financial cost, which is one of the main strengths of this study. Furthermore, because the intervention would be offered by the same nursing experts who work in the hospital every day, it would not result in major workload increases for professionals. Additionally, none of the participants expressed any concern or anxiety as a result of the online communication, indicating that the intervention did not add to the nursing staff's workload.

Although we observed the most anticipated improvements in GDM patients, our study has several limitations that warrant consideration. First, we did not evaluate the long-term prognosis of the two groups due to the short experimental period. Second, there was no follow-up after 37 weeks gestation, which should be considered in a future study. Third, a larger sample was not recruited since the study was presented to the hospital directors as an attempt to assess the feasibility and acceptability of the intervention in addition to the main measure of interest.

## Conclusions

To summarize, for patients with GDM, the internet combined with exercise-based individualized nursing intervention can effectively improve their GT and IR and significantly enhance their pregnancy outcomes and psychological state, which is worthy of clinical promotion.

## Data Availability

The datasets generated during and/or analyzed during the current study are available from the corresponding author on reasonable request.
